# Supports for Health and Social Service Providers from Canada Responding to the Disaster in Haiti

**DOI:** 10.1371/currents.dis.8821e785b58ec43043c7e46c82885409

**Published:** 2014-01-13

**Authors:** Christine Fahim, Tracey O'Sullivan, Dan Lane

**Affiliations:** Interdisciplinary School of Health Sciences, University of Ottawa, Ottawa, Ontario, Canada; Interdisciplinary School of Health Sciences, University of Ottawa, Ottawa, Ontario, Canada; Telfer School of Management, University of Ottawa, Ottawa, Ontario, Canada

## Abstract

In January 12, 2010, a 7.0 magnitude earthquake shook Port-au-Prince, Haiti. The massive disaster made it difficult for local Haitian community officials to respond immediately, leaving the country reliant on foreign aid and international and non-governmental relief organizations. This study explores the effectiveness of various supports that were made available to health and social service providers in Haiti, by focusing on their lived experiences pre-deployment, on-site and post-deployment. The paper provides a qualitative exploration of participant perceptions with respect to the success of their performance in response, and relevant literature describing the various supports provided to health and social service providers responding to disasters. Methods: A single, semi-structured interview was conducted with Canadian health professionals (n=21) who deployed to Haiti during the time of, or after, the 2010 earthquake. The study uses Strauss and Corbin’s structured approach to grounded theory to identify main themes and relationships in the interviews. Results: The interviews indicate that training, and psychological and emotional supports for health and social service providers require improvement to enhance the experience and effectiveness of their work. Conclusions: Findings indicate that supports are most effective when they are tailored to the volunteers. The paper highlights future research stemming from the grounded theory findings.

## INTRODUCTION

On January 12, 2010, a 7.0 magnitude earthquake struck Port-au-Prince, Haiti leaving 240,000 dead, 200,000 injured, 1.5 million homeless, and 600,000 displaced 1 ^,^ 2. The devastation was severe, destroying hospitals, clinics, and other infrastructure, leaving many health and social service workers buried in the rubble 3 ^,^ 4 ^,^ 5. To cope with the medical demands of its people, Haiti relied heavily on international relief^3^. While healthcare workers have repeatedly been identified as essential responders in the disaster relief effort, extant literature indicates they generally feel unprepared to respond to a large-scale emergency or natural disaster^6^. This leaves relief health and social service providers at physical, emotional and mental health risk, which could hinder their effectiveness as first responders. It is thus crucial for response organizations to ensure that responders are capable of performing their roles effectively without bringing harm to themselves or others^7^. There is a need to design systems for emergency preparedness that include adequate and appropriate supports for healthcare professionals involved in front-line disaster response. For the purposes of this paper, supports are defined as the various tools, provisions and programs available to health and social service providers responding to disasters. These tools provide responders with the physical, psychological and emotional resources to maintain resilience and effectiveness during deployment.

This study addresses support effectiveness and explores the various supports currently in place for health and social service workers providing humanitarian aid, while highlighting gaps in support, as perceived by responders themselves. Specifically, the purpose of this research is to understand and evaluate the types of supports provided to health and social service workers deployed to Haiti in response to the 2010 earthquake, and to learn from these experiences the means by which to design and improve support systems for foreign aid workers.

## METHODOLOGY

Participants were recruited from a variety of organizations including non-governmental organizations, universities, hospitals, the Canadian government, as well as faith-based organizations. Independent responders, and those in the military, were also interviewed, to provide contrast to the predominantly NGO-based sample and test the boundaries of the proposed findings. The inclusion criteria for the study required the individual to have served as a health or social service responder in Haiti during, or following, the 2010 earthquake. The research team interviewed health and social service responders deploying to Haiti up to 1 year after the disaster. Following university ethics approval, n=21 participants were recruited via email notices using purposeful and snowball sampling. The interviews took place between December 2011 and February 2012. The research team observed that many individuals responding within the first 3 months of the disaster were required to stay for a period of 2-3 weeks (which is considered a standard length of deployment through many large NGOs). To determine how the supports varied following the acute crisis of the earthquake, participants who deployed later in the year, as well as those who deployed for longer periods of time, were also recruited. These interviews were used to test the boundaries of the experiences of those who deployed immediately following the disaster in 2010.

Participant demographics are outlined in Table 1. Each participant was asked to participate in a single open-ended interview, 1 hour in duration, conducted by the primary author. The interview guide included questions pertaining to: training and preparation, systems and relationships in organized disaster response, stressors, roles, and the maintenance of an organized response system. A copy of the interview guide is attached in Appendix 1.

**Table 1: Participant Demographics:** 

*Org Type (n)*	*Professions*
NGO (n=10)	Physicians (n=7)Nurse (n=2)Program manager (n=1)
Faith-based Organizations (n=4)	Social worker/Psychologist (n=2)Program manager (n=1)Physician (n=1)
Independent (n=1)	Nurse (n=1)
Government/Military (n=5)	Program manager (n=2)Epidemiologist (n=1)Nurse (n=1)Physician (n=1)
Bilateral non-state institutions (e.g. universities) (n=1)	Physician (n=1)
TOTAL (n=21)	Physicians (n=10)Nurses (n=4)Program Managers (n=4)Epidemiologist (n=1)Social worker/Psychologists (n=2)

All data were transcribed, coded and analyzed using *NVivo9* qualitative software. The use of “thick descriptions”, detailed descriptions that present the context and emotion of the quote, were crucial to the validity of this study^8^. The study followed Strauss and Corbin’s 1990 approach to grounded theory. In accordance with this theory, the data were analyzed using inductive, open codes in order to identify emergent themes^8^^,^ 9 ^,^ 10. These categories were defined as:

1) *Contextual and Intervening Conditions*

2) *Causal Conditions*

3) *Strategies*

4) *Consequences* 10

The interview analysis process included the development of higher level pattern codes from the interview data to determine relationships between the categories and identify emergent themes. Tables 2 and 3 list all supports available to health responders. To determine which supports were most common, frequency scores for each individual type of support, by organization, were calculated. These tables were used as a supplement to the grounded theory analysis to highlight the supports available, as well as identify gaps in the individual supports.

**Figure:**
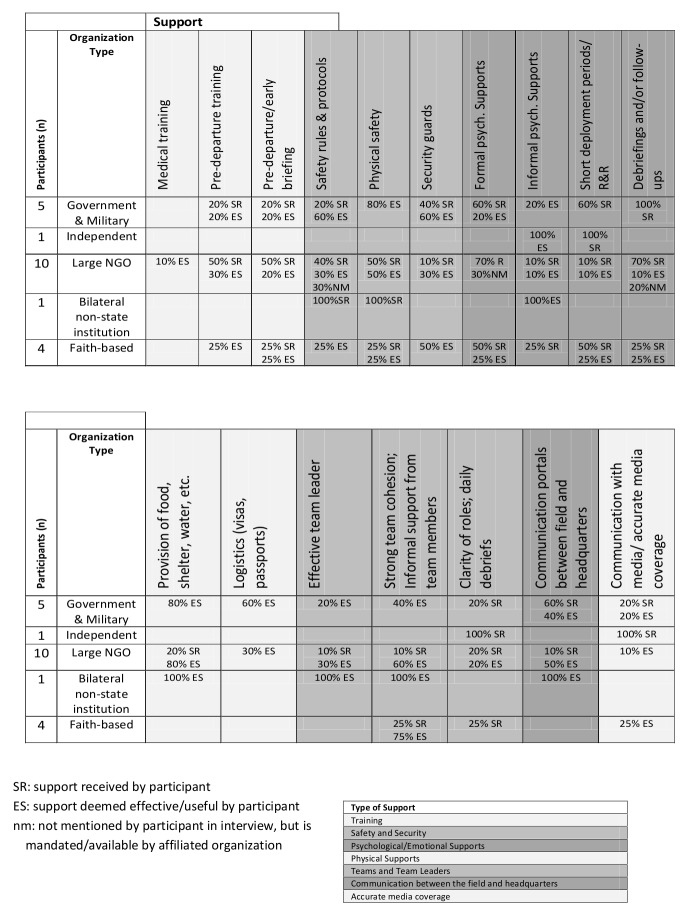
**Table 2: Supports received by participants**

**Figure:**
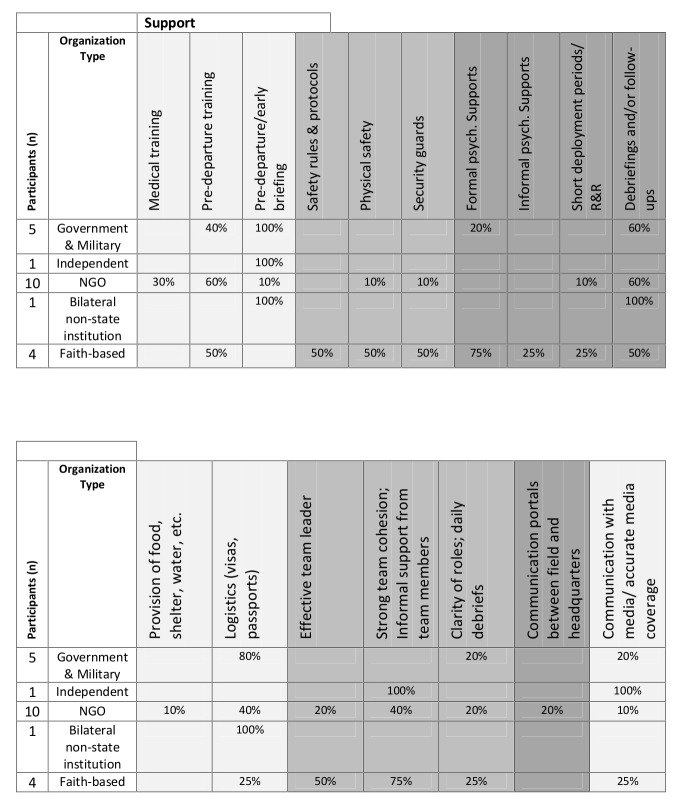
**Table 3: Participant recommendations on implementation/improvement of supports**

## RESULTS

*“Our first priority is not to our staff, as awful as that sounds, our first priority is to the work and to the end beneficiaries ...the staff care is important but often it comes second and third...when we get around to it...” (* *Canadian Government)*

The study results are summarized below according to the aforementioned categories. The central, or core, phenomenon of the study is identified as the *Need for Supports for Canadian Health and Social Service Providers who responded to the disaster in Haiti*, as well as the contextual, causal and consequential conditions that stem from these supports are illustrated in Figure 1. The *Context *refers to the societal, organizational and personal attributes and conditions that were present during the response. The first contextual factor, the country context, refers to the context within Haiti and the Haitian society at the time. Secondly, the context of the various relief organizations was explored to determine the organizational and coordinating factors that affected the response context. Finally, the personal context of the individuals responding to this disaster was analyzed.

**A Grounded Theory Model of the Context, Causes, Strategies and Consequences of the Need for Supports for Canadian Health and Social Service Providers Responding to the Disaster in Haiti (Fahim, 2012, adapted from Strauss and Corbin, 1990):**
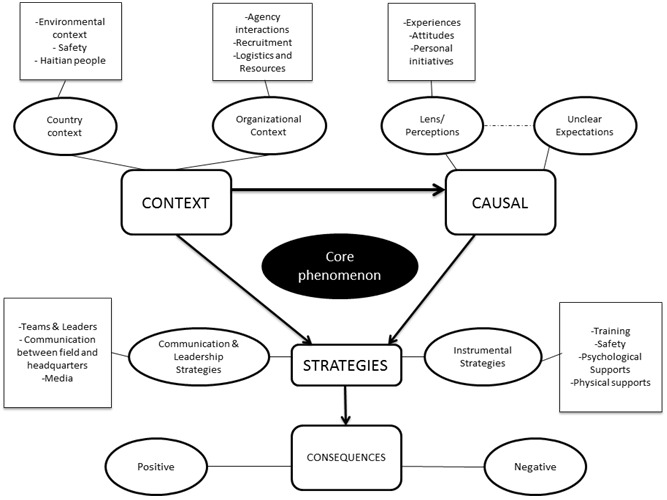

As seen in Figure 1, the *Causes *of the central phenomenon emerged and were integrated with the need for supports along two main themes. The first cause for support related to unclear expectations, which led to feelings of uncertainty and confusion, fear, lack of communication and awareness, and Post-Traumatic Stress Disorder. These feelings stemmed from a variety of causes, including unclear roles on-site, disorganization within teams and between organizations, and insufficient safety protocols and resources, training, and psychological and physical supports. The second main cause of the need for support relates to participants’ perceptions that influence their experiences, attitudes and personal initiatives. In response to the problems identified in the context and causes, organizations employed the use of various strategies to support their delegates. Analysis of the data resulted in the identification of two main categories of support in Haiti, Instrumental Supports, and Communication and Leadership Supports.

Finally, the *Consequences* of the response refer to the impacts, as perceived by the participants. These consequences were developed in relation to the context, causes and strategies, to highlight the strengths and weaknesses of the various supports present.

**Contextual and Intervening Conditions**

Already known as the ‘nation of NGOs’, the country context in Haiti was unstable prior to the earthquake, making the 2010 disaster both unique and challenging for responders. The earthquake created a barrier to food security, road access and phone communication, making it difficult for responders to assess the situation on the ground and report back to appropriate authorities. During the first few weeks after the earthquake, the ground was also unstable, leading to aftershocks that further destroyed the country’s already weakened infrastructure. As a result, responders were faced with the challenge of responding in a situation where they were forced to perform emergency procedures with little supplies, while coping with crumbling infrastructure, frequent tremors in the ground and harsh conditions, such as extreme heat. This context made logistical challenges a major frustration for the responders. Because much of the work was carried out in field-like hospitals, the logistical capacity of organizations fluctuated, depending on size and experience.

Upon arrival, many of the delegates witnessed a system in chaos, void of strong leadership of coordinating and government bodies, which had become compromised in the earthquake. Because there was no clear chain of command during the response, the initial result was disorganization and a lack of coordination. There were over 200 organizations that were registered in the Pan American Health Organization (PAHO) health cluster; however, project coordinators witnessed that there were few large organizations that had representatives and a strong voice at these ‘health cluster’ meetings. Some of the participants who attended the cluster meetings found the atmosphere to be, at times, hostile and uncoordinated. The notion of “egocentrism” came up repeatedly in the interviews. Larger organizations were described as territorial and preferring to work alone, perceiving smaller response organizations as peripheral. Some participants indicated there was pressure among independent volunteers, small NGOs, and faith based organizations to conform to the practices of larger organizations. ****Finally, the culture of the Haitian people also brought uniqueness to the country context. Many of the participants interviewed were amazed at the positive attitudes and resilient spirits held by Haitians during this difficult time.

**Causal Conditions**

The aforementioned context caused a need for a variety of physical and logistical supports. In addition to dealing with environmental challenges and crumbling infrastructure, many delegates fell sick while in Haiti, with some only recovering near the end of their deployment. Many participants stated that their roles were not clearly outlined pre-departure. Undefined roles often led to negative experiences, particularly when delegates felt their expertise was not being utilized. Some of the most commonly reported challenges included lack of clarity of roles, disorganization, and lack of leadership.

Finally, there was a need for psychological and emotional supports. One general attitude shared by many of the delegates was a feeling of ‘invincibility’. The study found that many healthcare professionals tend not to complain or ask for help, as they are accustomed to being ‘okay’ and being ‘the boss’. As a result, they were reluctant to admit when they were in need of emotional or psychological supports. This often led to stress, fatigue, frustration, and burn-out.

**Strategies**

The *Strategies* refer to the various supports that were provided to the responders in Haiti. These were classified into two major categories 1) Instrumental Supports and 2) Communication & Leadership Supports. The first of these categories, *Instrumental Supports*, refers to: (i) training strategies, (ii) safety and security supports, (iii) psychological and emotional supports, as well as (iv) physical supports. The second category, *Communication & Leadership Supports*, refer to (i) team dynamics and effective team leaders, (ii) communication between the field and headquarters, and (iii) communication with the media.

**Instrumental Support Strategies**

According to one delegate from a large NGO, the pre-deployment period to Haiti was very short. Some organizations chose to waive their typical two-week training period, due to the extent of damage from the earthquake, and the extreme need for immediate help. As a result, many delegates were sent to Haiti quickly, sometimes making the decision to leave within 24 hours. As a result, their training and briefing, particularly within the first few days, was not extensive. The interviews highlighted some forms of training that would have been an effective pre-departure strategy. The first of these was training during times of ‘normalcy’, to produce well-prepared responders that could be quickly deployed in an emergency. In addition, medical training would have been an effective pre-departure strategy, as many physicians and surgeons struggled to perform procedures uncommon to their specialty or rare to Canada. For the purposes of this paper, 'medical training' refers to training for specific medical procedures pertinent to the affected country (example: 'tenting' for cholera). Another type of training that was prominent in larger NGOs was Emergency Response Unit (ERU) training. This type of training provided responders with hands-on skills that allowed them to build and effectively run field-hospitals in a disaster zone. Larger organizations and NGOs, particularly those with previous experience in disaster response, seemed to be more regulated in their safety protocols. According to one participant from a large NGO,

“You make zero decisions about that [safety], you’re just told what to do by [organization name] and they’re quite rigid about that, as opposed to many other organizations where people are independent.” (NGO)

One of the most essential tools for maintaining safety was the provision of communication devices to ensure the location and safety of the delegate was not compromised. Other security supports included transportation in specialized vehicles. It was crucial that logistics and timing were accurate in order to avoid accidents and safety threats. Organizations needed to ensure that they were able to remove their delegates from the country if there was a need to do so.

There were many strategies available to address the third category of instrumental supports, focused on emotional and psychological needs. The context of Haiti made the need for these supports even greater. Supports varied greatly, depending on the individual’s organization, team leader and colleagues. One of the on-site strategies employed was the appointment of chaplains or counsellors who lived and worked with the delegates. These individuals were available to address any issues regarding stress or trauma experienced by the delegates, and often provided services in an ad-hoc fashion. Other organizations chose to employ on-the-spot critical incident clinical debriefings or morale and welfare officers when delegates had a difficult time with the country context or with patients. Program managers found that many responders did not ask for emotional and psychological supports. Other informal psychological and emotional health supports took the form of regular communication with family and loved ones back home. As stated by one participant,

 “For them [delegates] to be able to call their families...or just to hear their kids, was really important, so whoever you go with needs to be able to set up some kind of email or voiceover or satellite phone communication with home and make it available to their workers and their volunteers...if you go with a group that doesn’t have the infrastructure resources to set that up, it can be really tough.” (Faith-based organization)

The context of Haiti was very difficult, and many larger NGOs chose to utilize shorter deployment times, approximately 2-3 weeks, to protect their responders. Relief coordinators began to notice this trend, and attempted to adjust their deployment periods accordingly. When shorter deployment periods were not feasible, alternative strategies organizations chose to employ to combat burnout were rest and relaxation breaks (R&R). Some larger organizations provided delegates with the resources for respite in the Dominican Republic or Canada.

Finally there was the need to provide physical supports for the delegates. Larger organizations with greater experience in disaster response were typically well prepared to provide their delegates with the appropriate physical resources, such as food, water, and shelter.

**Communication and Leadership Support Strategies**

Participants stated the importance of having teams on site. As stated in the following participant quote,

“You go with this team and they become as close as your family when you’re there...they’re all you have and it’s amazing the relationships that develop, are like family.” (NGO)

One of the team strategies was the implementation of a ‘buddy system’. As stated by one nurse, “*make sure the novice, the rookies, have got a mentor.” *Some organizations chose to apply this technique pre-departure so the support would carry out both on-site and post-deployment. Organizations that were able to introduce delegates pre-departure found this to be an asset on-site, as complementary skills emerged within the teams. In addition to these more formalized strategies, there were ‘unwritten’ rules that were present on-site, such as being inclusive of new volunteers.

The second crucial element in response teams was the presence of an effective leader who possessed strong communication skills. In addition to providing encouragement and instruction, the team leader was responsible for ensuring that all of the delegates were aware of their roles and surroundings and were prepared to deal with emergency situations.

One of the strategies for effective communication included having lines of communication between individuals at headquarters and those in Haiti. Having individuals of equal rank and position communicate allowed for the establishment of clear expectations for those at headquarters and support for those on-site. As stated by a physician,

“Some administrator in Ottawa can tell me what they think I’m doing, a physician on the ground can tell me exactly what I’m doing. And there’s always a huge difference between what the head office thinks you’re doing and what the people on the ground know you’re doing.” (NGO)

Finally, accurate media coverage is very important to the relief response, as it is a driver of whether new delegates will volunteer to deploy to a disaster. The Haitian earthquake received extensive media coverage and attention. However, the situation portrayed in Haiti, particularly once the initial ‘response hype’ had settled, was often inaccurate and downplayed the efforts and effectiveness of the response, which led to much frustration among existing relief workers. **

**Consequences**

According to Strauss and Corbin (1990), *Consequences* are used to determine the effects of the central phenomenon^10^. In this study, the effects of the *need for supports* led to both positive and negative consequences. The *Consequences *outline the impacts of the various strategies, as perceived by the participants. Positive perceptions arose when an organizational strategy of instrumental or communication and leadership supports was tailored to an individual responder.

***Organizational Supports and Gaps.***

The interview information provided feedback from participants on their perceived gaps in organizational support. Table 2 is a summary of all of the supports by type received, and considered as particularly effective by the participants. These included Training Supports (medical training; pre-departure training; pre-departure/early briefing), Safety and Security Supports (safety rules and protocols; physical safety; security guards), Psychological and Emotional Supports (formal psychological supports; informal psychological supports; short deployment periods/rest and relaxation; debriefings and follow-ups), Physical Supports (provision of food shelter, water, etc.; logistic supports such as organization of visas, passports, etc.), Teams and Team Leaders (effective team leader; strong team cohesion and informal support from team members; clarity of roles and daily debriefs), Communication between the Field and Headquarters, Communication with the Media and Accurate Media Coverage. Support received, or “SR” was placed next to every support cited in the participant interviews. If a participant found the support to be particularly effective, the mark was replaced by “ES” for effective support. Finally, “NM” or not mentioned, was placed in the table if the organization was known to provide a particular support, yet the support was not mentioned in the interview. An overall tally of each provided support was calculated, by organization, in order to distinguish the supports most frequently provided to the responders.

According to the data, 67% of participants (n=14 of 21) were given training supports, safety and security supports were provided to 86% of the delegates (n=18 of 21), and some form of psychological and emotional support was provided to 100% of the participants. Physical supports were provided to 81% of the participants (n=17 of 21) and 76% of participants (n=16 of 21) reported having been given team supports and had effective team leaders. Finally, 57% of participants (n=11 of 21) were provided with communication portals between the field and headquarters and 24% (n=5 of 21) reported effective communication with media and accurate media coverage.

Table 3 presents a summary of all the supports the participants considered needed improvement and were thus deemed insufficient. The need for improved training supports was the biggest identified gap (71% of participants). In particular, participants highlighted the need for improved pre-departure training and briefing. Improved psychological and emotional supports were also strongly identified as a need by 62% of participants. In particular, the need for debriefings and follow-ups were recommended by 57% of the participants. Another support strongly recommended was the need for logistical support, including organization of visas, passports, etc. Finally, 57% of participants expressed a need for improved teams and team leadership. The consequences of some of these supports are highlighted below.

***Consequences of Instrumental Support Strategies*** *.*

**Participants found that training during times of normalcy was very effective, in contrast to organizations that waited until the disaster struck to train their delegates. This left a very small window of opportunity for effective training, thereby increasing stress and disorganization. As one participant states,

“*It’s very hard to show someone what to expect even though you try, so it’s a difficult task to brief someone...people can only absorb so much when they come down...it doesn’t translate to being well prepared when you come.” (Faith-based organization)*

The second type of instrumental support that was provided by organizations was safety and security. Most delegates were comforted by the presence of safety personnel and high security, and those who deployed with organizations that did not provide these supports cited this as a gap. However, some delegates who entered the response with background experience in disaster response, and who were in Haiti for prolonged periods of time, eventually found these security supports to be stifling and recommended that organizations find a balance in their safety provisions. One program manager recalls,

“*I know volunteers...some of them were a little shocked at the level of security and wondered ‘why do we have so much security, is there something we need to be worried about here?’ And that got them more upset. And then some of them were really relieved to see all of the security.” (Faith-based organization)*

The third instrumental strategy of support was psychological and emotional supports. The data indicated that responders had a hard time accepting support from mental health personnel, unless they had developed a personal rapport with them. Many responders shared an attitude of “invincibility”, which amplified the need to develop strategies that would allow delegates to utilize psychological supports without feeling stigmatized as ‘psychologically weak’ or ‘in *need* of help’. Team dynamics and emotional support from colleagues were also crucial. As stated by one participant who spoke about using her husband for emotional support regarding her experience with Katrina,

“I quickly learned that wasn’t a good idea because my husband worried about me. He didn’t know that this [anxiety] was normal and that it would pass...he just wanted to make it go away and his response was ‘you shouldn’t do this stuff anymore’. So I learned not to do that, because it’s not fair to him either, and then you also bring up the issue of secondary traumatisation.” (Faith based organization)

She stated the importance of having a friend on-site who was able to relate to the experiences and provide her with that much needed support.

A final strategy that was used to promote psychological and mental health was to have defined periods of deployment. Shorter periods of deployment were recommended to those who responded immediately after the disaster. The organizations were faced with choosing a deployment period that was long enough to allow for effective work and continuity, but short enough to ensure that personnel did not burn out whilst in Haiti. This dilemma was highlighted by a few of the participants,

“Some [delegates] definitely should have gone home...that’s the catch 22...ten days wasn’t enough to do what you wanted to do. Two or three weeks would have been optimum...but I can see how healthy it would be for people to take a break after two weeks even if it’s heading back to North America for a week and then re-deploying” (Faith based organization)

Upon completing their deployment, many participants experienced grief and had difficulty resuming their normal routines. Much of this was attributed to feelings of guilt for leaving the disaster situation, combined with fatigue.

“I went back to Canada for a reprieve...I get into the airplane and I look back and I saw the unfinished work and suddenly I had a feeling as if I’m abandoning those people and it was awful. So I spent the whole time flying from Haiti to Ottawa crying, because [I’d] abandoned them” (Bilateral non-state institution)

Finally, many of the participants shared the common difficulty of returning to their demanding vocations shortly after their return. One of the organizational strategies used to address these feelings of stress was debriefing and follow-ups. Many participants believed that follow-ups not only allowed them the option of emotional support, but gave organizations the opportunity to improve, learn from their mistakes, and address any gaps in the supports that were provided. These participants recommended that debriefings take place immediately upon return. As stated by one participant,

“Most people probably say ‘I don’t need to’ [debrief] but it might be a precaution...when there’s so much death and destruction around you...something like that should be made probably mandatory.” (Canadian Government)

The final type of instrumental strategy was the provision of physical supports. Many participants who deployed with smaller organizations recalled having to bring all of their own medical equipment, drinking water and resources to Haiti. Participants who did not have logisticians to organize physical resources and provide guidance and support to medical best practices reported feelings of frustration.

**Consequences of Communication and Leadership Strategies**

Team dynamics were a critical element of the response. Effective team leaders ensured delegates were provided with a clear sense of purpose and defined roles and tasks, to avoid confusion and frustration. As stated by a participating surgeon,

 “The day I arrived, they informed me a) there would be no anaesthetist available, and b) there would be no elective surgery to be performed, so the day I got there, they told me that they didn’t need me. I ran out of patients, saw people and advised them...but we didn’t do nearly as much surgery as we could have.” (NGO)

Another strategy that was important to delegates was the importance of communication between those on-site and headquarters in Canada. Lack of communication is stressful not only to the delegates on-site but to those working in headquarters. As stated by a program manager,

“...all the communications were shut down and it wasn’t possible to contact the [on-site] people in our team...from a head office perspective, it was very hard for us not knowing if they were alive or if they were dead, so it was a very stressful situation.” (NGO)

## DISCUSSION

According to our results, 71% of participants identified a need for improved training supports, particularly pre-deployment training and briefing, making this the biggest identified gap in this study. The literature echoes these findings, indicating that there is currently no universal standard when it comes to the availability and usage of standard training guidelines, making training supports highly varied, dependent on the affiliated organization^11^. To avoid overwhelming responders with on-site training information, participants suggested holding pre-deployment online modules and training sessions. The interviews indicate that these sessions should be held pre-disaster, during times of ‘normalcy’, and should encompass a variety of topics, such as information on the political and economic situation of the country, roles and team dynamics. In addition, pre-departure and on-site training modules should prepare delegates to deal effectively with the context, roles, security threats and psychological risks on-site, in order to avoid discouragement, panic and burnout^12^^,^ 13 ^,^ 14 ^,^ 15. Finally, training supports for health professionals should also include medical training, with focus on practical skills and tools required to respond in resource-limited settings^16^^,^ 17 ^,^ 18.

Findings show that while all of the participants reported being provided with some form of psychological and emotional support, it was identified as one the least effective supports. This study supports the literature that suggests that psychological and emotional supports are crucial at the pre-departure, on-site and post-deployment phases of the response^14^^,^ 19 ^,^ 20. According to the participants, the most effective forms of this support included the integration of mental health workers in the response team, and strong teams and team leadership. The importance of effective communication and trust within teams was found to be crucial in a disaster response, particularly in a context such as Haiti. As seen in the literature and the interview data, an individual’s performance and outlook are shown to be affected by the attitudes, initiatives and goals of their team members^12^^,^ 13 ^,^ 14 ^,^ 21. As a result, appointed team leaders should possess a positive, encouraging attitude, to support resiliency among their colleagues. Effective team leaders reminded delegates of their goals, held daily debriefs, paired novice volunteers with seasoned responders, allowed time for communication with loved ones back home, and provided responders with space and time to share their experiences and concerns.

Fifty-seven per cent of participants in this study indicated they would have liked a debriefing or follow up almost immediately upon return, which differs from some of the popular literature. According to Suzuki, Nakajima & Kim (2012), “debriefing should not be conducted immediately after disasters because of the risk of making [delegates] relive the experience. Some even get hurt after talking about work that they should have done” (p.18). They recommend that the supports be made available for when a delegate is willing to have this conversation^22^. However, as seen in the findings of this study, most health and social service providers do not *willingly* use or seek out these supports. Therefore, while organizations should be careful not to pressure responders to disclose information, the recommendation is that program managers appoint licensed, experienced psychologists to place follow-up phone calls to delegates upon their return. This follow-up should be used as an opportunity to thank responders and make them aware of any supports available to them post-deployment.

Finally, organizations should recognize the importance of being self-sufficient and ensure that the details of their responders’ deployments are well planned. Organizations that were found to be the most resilient in Haiti typically provided their delegates with field hospitals and housing. They also highlighted the importance of logistics, such as flights, transportation, visas, passports, vaccinations. Finally, these, typically larger, organizations, often had the capacity to allocate budgets for food and water for responders, equipment repair, and emergency medical support for responders.

## CONCLUSION

These findings open the door for future researchers to explore the individual components of each of the support categories and further describe the relationships provided in the model. Primarily, researchers should investigate the identified supports that were provided to the responders in Haiti, using the results and consequences to improve gaps in the current systems of support.

## LIMITATIONS

This study has certain limitations that should be acknowledged. The demographic breakdown of the participants was not equally representative by occupation. A large number of participants in the sample were physicians (n=10) or from large organizations (n=10), which may have affected the findings. Future researchers should use the results of this preliminary study to conduct a follow up study, using a bigger sample size, which would allow organizations to better direct their focus according to their volunteer demographic and generalize findings. Secondly, it is important to remember that Haiti was an extensive disaster with a unique context. Regardless, all of the identified supports can be tailored to suit the particular context of the emergency, as well as the needs of the responders.

## Appendix 1

### Interview Guide

**Demographic Information (to be collected before the interview)          **

Affiliated Organization: ________________________________________________________

Occupation: ________________________________________________

Years of Experience

    1-5 years

    6-10 years

    10-20 years

    20+ years

Credentials/Level of Education: _________________________________________

Date of Departure to Haiti: _________________________________________

Have you responded to any other disasters?

      If yes, please list: _________________________________________

Did you have any dependents (ie: spouse, children) at the time of departure? If yes, please check the box(es) that apply.

    Spouse

    Child(ren)

    Parent

    Other

**Interview Questions**

1. Could you walk me through the steps that you went through with [affiliated organization] pre-deployment?

Probe: Were any supports provided to you through [affiliated organization] pre-deployment?

Probe: Were these supports effective? Please explain.

2. What were your expectations going into the response?

3. What were your motivations/reasons for choosing to response to this disaster?

4. Could you tell me a little bit about your role in the Haitian disaster response?

Probe: What was the chain of command that you witnessed? (if any)

5. Did you work with a team during your time abroad?

Probe: What type of team? (ie: Interdisciplinary, health professionals)

Probe: Could you describe to me the culture of this team? (ie: team dynamics)

6. Could you please describe your experiences in Haiti during the time of the response?

7. Did you feel prepared to respond to this disaster situation?

8. Were you provided with any supports during your time in Haiti?

 Probe: Could you please describe these?

Probe: Were they effective?

Probe: Who provided these supports?

Probe: Were these supports available to everyone? (just relief workers, just healthcare professionals, just individuals from [affiliated organization])

9. In your opinion, were you given the appropriate resources to perform your role effectively?

Probe: If yes, please tell me more Probe: If no, what was missing?

10. Please describe your experiences returning home after the disaster

Probe: Were you able to fall back into your routine upon return? Why or why not?

Probe: Did you feel that your expectations of the response were met?

11. Did you feel that there were any gaps in the types of supports that were available to you? If so, please describe these gaps.

12. If you could design the best type of training and support systems for someone like yourself going abroad, what would these look like?

That’s the end of my questionnaire. Is there anything else that you would like to highlight/address?
